# The distribution of the apparent diffusion coefficient as an indicator of the response to chemotherapeutics in ovarian tumour xenografts

**DOI:** 10.1038/srep42905

**Published:** 2017-02-21

**Authors:** Monique C. Tourell, Ali Shokoohmand, Marietta Landgraf, Nina P. Holzapfel, Patrina S. P. Poh, Daniela Loessner, Konstantin I. Momot

**Affiliations:** 1 Queensland University of Technology (QUT), Brisbane, Queensland (QLD), Australia; 2 Australian Prostate Cancer Research Centre - Queensland, Translational Research Institute, Brisbane, QLD, Australia; 3 Experimental Trauma Surgery, Department of Trauma Surgery, Klinikum rechts der Isar, Technical University of Munich, Munich, Germany

## Abstract

Diffusion-weighted magnetic resonance imaging (DW-MRI) was used to evaluate the effects of single-agent and combination treatment regimens in a spheroid-based animal model of ovarian cancer. Ovarian tumour xenografts grown in non-obese diabetic/severe-combined-immunodeficiency (NOD/SCID) mice were treated with carboplatin or paclitaxel, or combination carboplatin/paclitaxel chemotherapy regimens. After 4 weeks of treatment, tumours were extracted and underwent DW-MRI, mechanical testing, immunohistochemical and gene expression analyses. The distribution of the apparent diffusion coefficient (ADC) exhibited an upward shift as a result of each treatment regimen. The 99-th percentile of the ADC distribution (“maximum ADC”) exhibited a strong correlation with the tumour size (r^2^ = 0.90) and with the *inverse* of the elastic modulus (r^2^ = 0.96). Single-agent paclitaxel (n = 5) and combination carboplatin/paclitaxel (n = 2) treatment regimens were more effective in inducing changes in regions of higher cell density than single-agent carboplatin (n = 3) or the no-treatment control (n = 5). The maximum ADC was a good indicator of treatment-induced cell death and changes in the extracellular matrix (ECM). Comparative analysis of the tumours’ ADC distribution, mechanical properties and ECM constituents provides insights into the molecular and cellular response of the ovarian tumour xenografts to chemotherapy. Increased sample sizes are recommended for future studies. We propose experimental approaches to evaluation of the timeline of the tumour’s response to treatment.

Diffusion-weighted magnetic resonance imaging (DW-MRI) is a well-established technique for quantitative evaluation of high-cellularity tumours in both clinical and research settings. The apparent diffusion coefficient (ADC) measured from DW-MRI is sensitive to the tumour microenvironment. As a result, the ADC is a potential non-invasive biomarker for the initial identification of tumour masses, as well as prediction and monitoring of response to therapy^1^,^2^,^3^,^4^,^5^,^6^.

ADC is negatively correlated with the tumour cellularity, with high cell density and extracellular tortuosity resulting in increased restriction on the diffusion of water molecules^7^,^8^,^9^. Conversely, apoptotic or necrotic areas of tumours are associated with elevated ADC values as a result of decreased cell density^10^,^11^,^12^, while high-cellularity tumours may exhibit significantly decreased ADC values^13^,^14^,^15^,^16^.

ADC values in tumours are also known to increase in response to anti-cancer therapy^17^,^18^,^19^. A lower number of proliferating cells and a higher number apoptotic cells are indicators of therapy success. The decrease in cell density, as well as structural changes that precede or accompany cell death (*e.g.* cellular shrinkage during apoptosis or membrane rupture during necrosis), lead to reduced restriction of water diffusion in both intra- and extracellular spaces. The subsequent increase in ADC values has been observed in tumour xenografts^20^,^21^,^22^,^23^,^24^,^25^,^26^ and patient-derived samples^27^,^28^,^29^,^30^,^31^ for a wide variety of tumour entities and therapeutic regimens. Some of these studies have included treatment using platinum-26,30,31 and taxane-based^20^,^29^,^30^,^31^ chemotherapeutics. Besides mean ADC values, the changes of ADC distributions in response to therapy have also been investigated and found to be a more comprehensive indicator of this response than the mean ADC alone^30^.

Combination platinum/taxane-based chemotherapy is the standard first-line therapy in the clinic for the treatment of patients diagnosed with ovarian cancer^32^. The individual effect of single-agent platinum or taxane and combination platinum/taxane treatment on the ADC in ovarian tumour xenografts has not yet been determined. However, their general effect has been studied implicitly in clinical research. Kyriazi *et al*.^30^ and Sala *et al*.^31^ reported a 16% and 28% increase in the mean ADC of water in platinum- or taxane-responsive ovarian tumours, respectively.

In the present study, we examined the effect of different chemotherapy regimens, single-agent platinum and taxane and combination platinum/taxane, on the ADC distributions in ovarian tumour xenografts derived from human serous ovarian cancer cells, using diffusion-weighted magnetic resonance microimaging. Microimaging enables enhanced spatial resolution compared to clinical MRI, and can provide information on the local microscopic environment and macroscopic organisation of the sample. We observed changes in the ADC distributions of the tumours as a result of each of the treatment regimens. We discuss these observations in terms of the relationship between tumour response to chemotherapy and the production of extracellular matrix (ECM) and propose future experiments that would provide insights into the detailed time line of this response.

## Methods

### Animal Study

The animal experiment was performed in compliance with the Australian Code of Practice for the Care and Use of Animals for Scientific Purposes and approved by the University Animal Research Ethics Committees (Queensland University of Technology approval 0900000578; University of Queensland approval QUT/287/11). Surgery using 6-week old female NOD/SCID mice was performed as described previously^33^. Polyethylene glycol-based hydrogels were used as an ovarian cancer OV-MZ-6 cell spheroid delivery vehicle. These were implanted intra-peritoneally between the ovarian bursa and nearby fat tissue, adjacent to both ovaries. Tumour growth was assessed weekly via bioluminescence as reported earlier^33^. To examine therapeutic effects of platinum/taxane treatment on tumour growth, treatment started 4 weeks post-implantation according to our previous study^33^. The treatment regimen consisted of either carboplatin (Sigma-Aldrich, Castle Hill, NSW, Australia; 15 mg/kg/dose), or paclitaxel (Sigma-Aldrich; 10 mg/kg/dose), or a combination of both chemotherapeutics, given twice per week each in an alternating manner for all treatment groups, via intra-peritoneal injections for 4 weeks (5 mice per group). Saline or DMSO served as control treatment. Eight weeks post-implantation, animals were sacrificed, and tumour tissues were removed and subjected to DW-MRI, mechanical testing, immunohistochemical and gene expression analyses.

### DW-MRI

Fresh, non-fixed tumour tissue samples were placed on top of a custom-made Teflon plug positioned at the bottom of a 10 mm NMR tube (Wilmad) in phosphate-buffered saline (PBS). Samples thus prepared and refrigerated can be stored for at least a week, as shown in our previous study^35^. A second Teflon plug was positioned above the sample, allowing ~5 mm of PBS between the samples and top plug for calibration of the diffusion data. The total distance between the two Teflon plugs ranged from 10 to 15 mm. MRI measurements were performed at room temperature on a Bruker Avance NMR spectrometer with a 7.0 T vertical bore superconducting magnet equipped with a Micro2.5 micro-imaging probe and a triple-axis gradient set (maximum gradient amplitude 1.2 T/m)^34^. A 10 mm birdcage RF coil was used. For all measurements, the imaging plane passed through the centre point of the NMR tube and was vertical (aligned with the long axis of the NMR tube and **B**_**0**_). The Read direction was in the direction of the **B**_**0**_ field.

A Fluid-Attenuated Inversion Recovery (FLAIR) spin-echo imaging sequence was initially used to locate the position of the samples in the NMR tube and to determine the appropriate imaging plane for the diffusion measurements. FLAIR parameters were similar to those reported in our previous study^35^. The duration of the inversion recovery interval was empirically chosen as 1.4 s in order to minimise the PBS signal. From these images, the azimuthal orientation of the vertical imaging slice for the diffusion imaging was chosen such as to contain the largest cross-section of the tumour tissue sample.

Diffusion imaging was performed using a spin-echo diffusion-weighted pulse sequence with TR = 3 s, TE = 14 ms and 3-lobe sinc RF pulses (1 ms duration for the excitation 90° pulses and 0.749 ms for the refocusing 180° pulses). Slice thickness was selected to accommodate the sample size. For the control, single-agent carboplatin and paclitaxel treatment groups, the slice thickness was set to 0.4 mm; for the combination carboplatin/paclitaxel treatment group, to 0.3 mm. In-plane imaging parameters were: FOV = 19.8 mm (Read) × 14.9 mm (Phase), NR × NP = 170 × 128 (zero-filled to 256 × 128). The diffusion gradient direction was Read. The diffusion-weighing gradients had the duration δ = 2 ms, with the gradient separation (diffusion interval) Δ = 7 ms. The largest gradient amplitude used was 63% of the rated maximum amplitude (*g *≤ 0.76 T/m). For each group, 8 diffusion-weighted images were acquired with *b* values equally spaced from 0 to 1400 s mm^−2^. The linearity of the Stejskal−Tanner plots (checked for a representative selection of voxels from both the tumour and the PBS regions) was used to ensure the absence of thermal convection or mechanical vibration effects.

### Mechanical Testing

Unconfined mechanical compression testing of fresh, non-fixed tumour tissue samples was conducted using an Instron 5848 micro-tester fitted with a 5 N load cell at 37 °C, with PBS as the immersion medium as described previously^35^. Samples were subjected to 30% compression relative to the uncompressed height at a rate of 5 mm per minute, and the Young’s modulus was determined at 25% strain using the stress versus strain data set.

### Immunohistochemistry

Haematoxylin/Eosin (H&E) staining and immunohistochemical analysis were performed on serial paraformaldehyde-fixed paraffin-embedded tumour tissue sections (5 μm), from the same tumour tissue samples as used for DW-MRI. H&E staining was conducted to visualise tissue morphology using a standard procedure as reported previously^36^. For immunohistological analysis, samples were deparaffinised in xylene and rehydrated in dilutions of ethanol and water. Antigen retrieval was performed using a high-pH buffer (pH 9) at 95 °C for 10 min. Then, samples were treated with 3% H_2_O_2_ and blocked with 2% bovine serum albumin (BSA)/PBS. The human-specific antibodies against the nuclear mitotic apparatus protein 1 (NuMA; Epitomics, Burlingame, CA, USA) and Ki67 (#MIB-1; Dako, Sydney, NSW, Australia) were applied 1:100 and 1:75, respectively, in 2% BSA/PBS as reported previously^36^. After washing, sections were incubated with EnVision+Dual Link System-horseradish peroxidase (HRP; Dako), followed by 3,3′-diaminobenzidine chromogen (Dako) and Mayer’s haematoxylin (Sigma-Aldrich) staining. Sections were imaged using an automatic Leica slide scanner with a 40x magnification and archived on a digital image hub. Quantification of Ki67-stained sections was performed using ImageJ (NIH, Bethesda, MD, USA), and the percentage of the positively stained tissue area was calculated.

### Calculation of ADC

A custom in-house *Mathematica* code (Wolfram Research, Champaign, IL, USA) was used for the analysis of all MR images. A region of interest (ROI) containing voxels of only PBS was identified in the diffusion-weighted images and used to calibrate the effective *b*-values as described previously^35^. The calibrated *b*-values were then used to construct the apparent diffusivity maps for each treatment group by constructing a linearised Stejskal−Tanner diffusion plot and performing a linear least-squares fit of the signal decay in every ROI voxel. The tumour ROI for ADC analysis was determined by inspection of the ADC maps thus constructed, with reference to FLAIR, diffusion-weighted MR and immunohistochemical images. Excluded from the ROIs were regions of murine muscle and fat tissues and tumour regions that had been infiltrated by surrounding murine tissue. Additionally, tumour regions near sutures (easily identified in the reference diffusion images acquired with *b* = 0 s mm^−2^) were avoided. Histograms of the diffusivities for each treatment group were constructed from the tumour ROIs. The distributions were compared using the pairwise Mann-Whitney test.

### Measurements of Tumour Area

The histological area of tumour tissue sections using only the NuMA-positive tumour regions was calculated using ImageJ. The cross-sectional area of the tumours visible in the MRI analysis was calculated by drawing a ROI around the solid tumour tissues. This region was typically *not* identical to the one used to construct the ADC map. The uncertainty in these areas was calculated by adding or subtracting a layer, one pixel wide, to the perimeter of the regions. The longest axis of each separate, solid mass visible in the MR image was also measured. The uncertainty in each measured length was the length of two pixels.

### Gene Expression Analysis

Tumour tissues were dispersed with a tissue homogeniser, and RNA extracted using Trizol^®^ reagent (Life Technologies, Mulgrave, VIC, Australia) following the manufacturer’s instructions. RNA quality was determined with a Nanodrop^®^ ND-1000 spectrophotometer (Life Technologies), and samples with A_260nm/230nm_ = 2.05–2.15 were synthesised into cDNA using a Superscript™ III first-strand synthesis supermix (Life Technologies) following the manufacturer’s instructions. Quantitative RT-PCR was performed using SYBR^®^ green chemistry using an ABI Prism^®^ 7500 sequence detection system (Life Technologies) as reported previously^33^. Gene-specific primer sets are listed 5′-3′, forward and reverse respectively: *FN1* GTGTGACCCTCATGAGGCAAC and CTGGCCTCCAAAGCATGTG, *VTN* TGCTGGCATGGGTTGCT and GTTCATGGACAGTGGCATTGTT, *COL1A1* GTGCTCCTGGTATTGCTGGT and ACCAGGTTCACCGCTGTTAC, *HIF1A* GGGTTGAAACTCAAGCAACTGTC and GTGCTGAATAATACCACTCACAACG, *18 S* GATCCATTGGAGGGCAAGTCT and CCAAGATCCAACTACGAGCTTTTT. The cycle threshold (C_t_) of each gene of interest was determined and normalised to *18 S* expression levels and compared to the control (ΔΔC_t_). Relative gene expression between treatment groups was calculated using the 2^−ΔΔC_t_^ method.

## Results

### Immunohistochemistry

**Figure 1:**
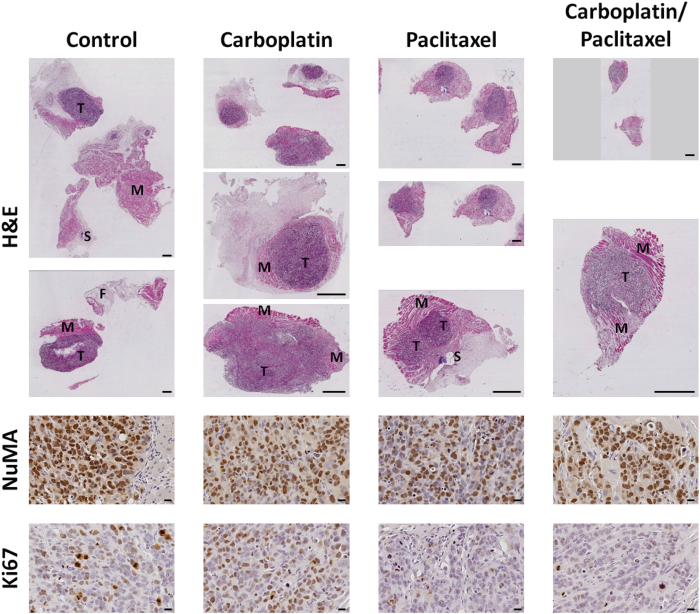
H&E staining and immunohistochemical analysis of the different treatment groups. Tissue morphology is indicated by H&E staining as an overview and close-up of the tissue samples (scale bars, 1 mm; T – tumour tissue, M – muscle tissue, F – fat tissue, S – suture). Paclitaxel-containing treatments resulted in smaller tumour regions compared to single-agent carboplatin and the control (no treatment). Representative images of nuclear NuMA and Ki67 stainings are indicated in brown for all groups, with the control and single-agent carboplatin groups showing a higher number of proliferating cells (scale bars, 20 μm).

H&E staining of the complete set of the xenograft samples from all the tumour tissues analysed by DW-MRI has revealed the morphological heterogeneity of the tissues. At the tumour margins, murine muscle and/or fat tissues were apparent (Fig. 1, top). NuMA staining enabled specific identification of the tumour tissue areas, with large tumours in the control group, and small or minimal tumour regions in all treatment groups (Fig. 1, middle). Ki67 staining indicated that cell proliferation was reduced in treatment regimens containing paclitaxel compared to the control and single-agent carboplatin treatment groups (Fig. 1, bottom). Quantitative image analysis of the percentage of the positively stained tissue area revealed a significant reduction of proliferating cells upon single-agent paclitaxel (0.31 ± 0.05; P < 0.001) and combination carboplatin/paclitaxel (0.23 ± 0.07; P < 0.001) compared to the control (3.03 ± 0.63), while the single-agent carboplatin group (3.45 ± 0.64) displayed a similar amount of Ki67 positive cells as seen in the control group.

### DW-MRI

**Figure 2:**
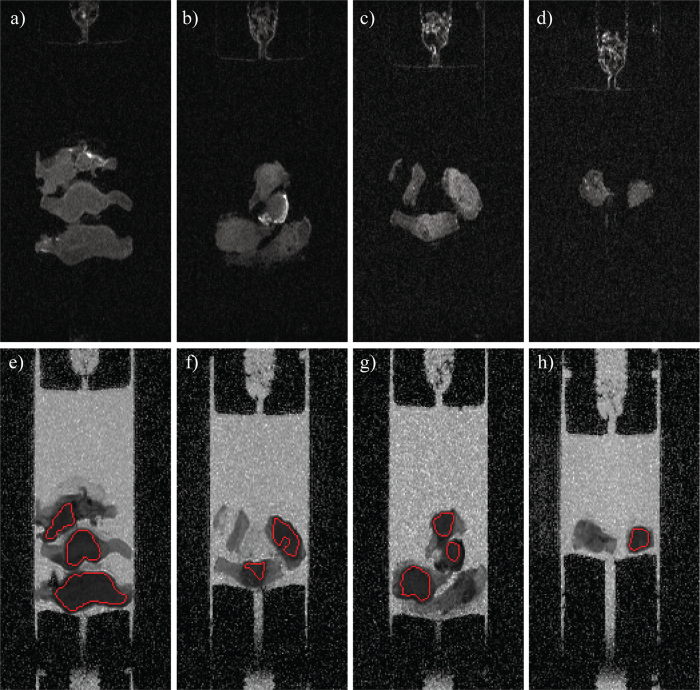
FLAIR images (top row) and ADC maps (bottom row) for the different treatment groups: (**a**) and (**e**) control (no treatment); (**b**) and (**f**) carboplatin; (**c**) and (**g**) paclitaxel; (**d**) and (**h**) combination carboplatin/paclitaxel. The red lines in the ADC maps indicate the ROIs used for the histogram analysis.

FLAIR images for each treatment group (Fig. 2a–d) indicated that the tumour and the murine muscle tissues were not clearly distinguishable on the basis of relaxation-weighted images alone. Murine fat tissue, predominantly observed in the control and carboplatin treatment groups (Fig. 1), had a relatively short *T*_1_ compared to PBS and the muscle tissue, and appeared hyper-intense in the FLAIR images (Fig. 2a,b). Solid tumour regions were identified from the ADC maps constructed for each of the different treatment groups (Fig. 2e–h), with reference to the H&E and histological images and the calculated ADCs. Tumour regions appeared as darker grey areas in the ADC maps, while lighter grey areas in the ADC maps, often surrounding the tumour tissue, were identified as murine muscle tissues.

### ADC

**Figure 3:**
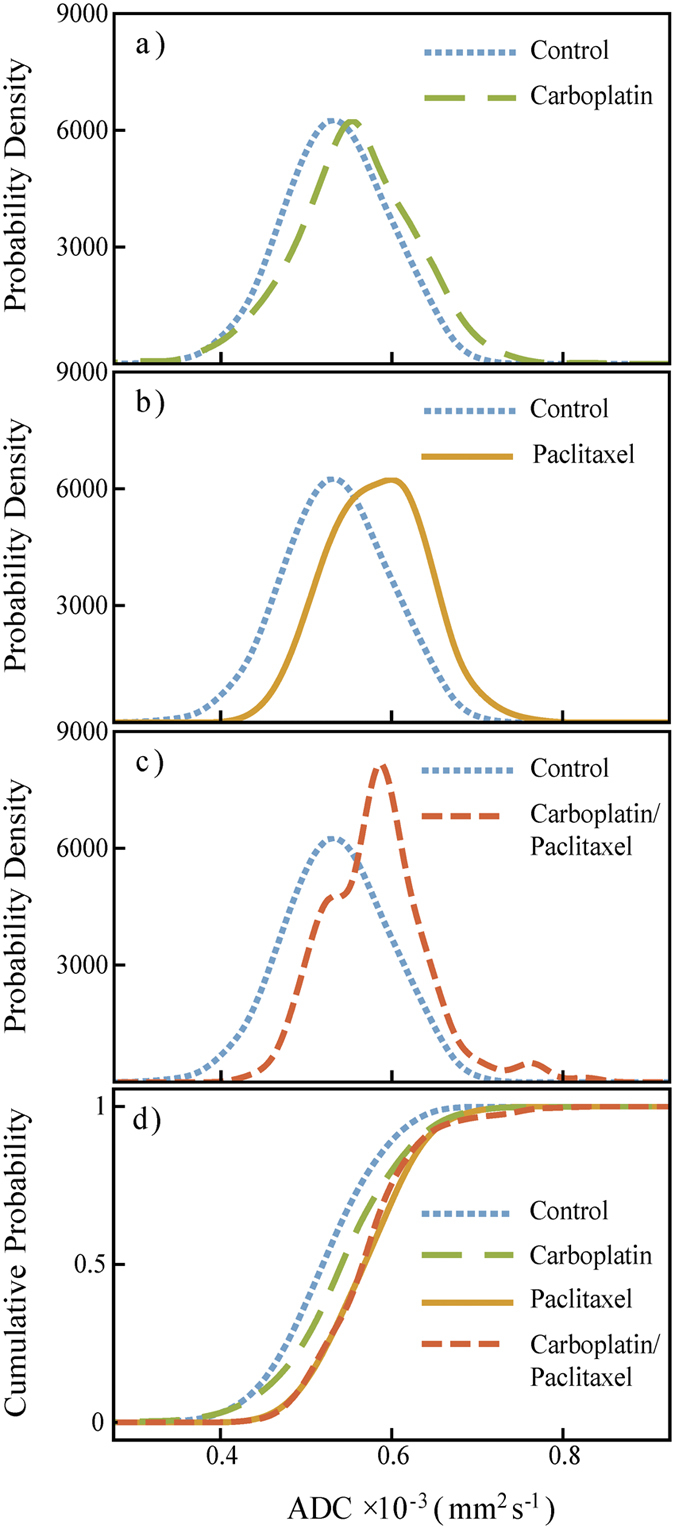
Comparison of the ADC distributions in the treatment and the control groups. The blue dotted line corresponds to the control (no treatment) group and is displayed together with each of the treatment groups: (**a**) carboplatin (green line); (**b**) paclitaxel (orange line); (**c**) combination carboplatin/paclitaxel (red line). The cumulative probability plots of all four ADC distributions is shown in (**d**). For clarity, only the splines of the distributions are shown.

The ROIs used to calculate the ADC distributions for each of the treatment groups are indicated by the red lines in Fig. 2e–h. The ADC distributions of the single-agent carboplatin, paclitaxel and combination carboplatin/paclitaxel treatment groups are shown next to the control group in Fig. 3a–c. A quantitative summary of each ADC distribution is given in Table 1. The ADC distribution of each treatment group was found to be significantly different (α = 10^−4^) from the control group on the basis of the pairwise Mann-Whitney test (see Table 2). For all treatment groups, the ADC distributions exhibited a shift towards higher ADC values compared to that of the control group (Fig. 3a–d, Table 1). The change in ADC values from the control group was non-uniform and dependent on the treatment regimen. The mean ADC value of the single-agent carboplatin group was significantly different from the single-agent paclitaxel and combination carboplatin/paclitaxel mean ADC values (Table 2). However, there was no statistically significant difference between the ADC distributions of the single-agent paclitaxel and the combination carboplatin/paclitaxel treatment (Table 2).

### Tumour Area

**Figure 4:**
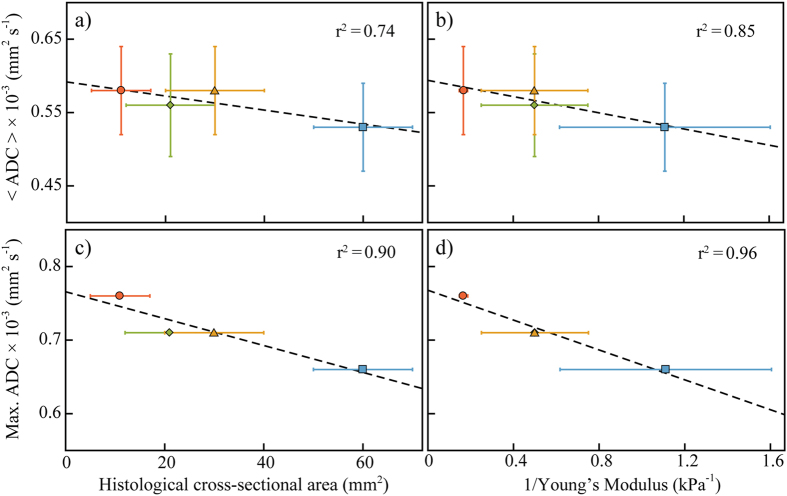
The mean ADC, < ADC >, (**a**) and (**b**), and the maximum ADC, (**c**) and (**d**), correlated with tumour cross-sectional size and elastic modulus, respectively. The black, dashed line in each plot is the least squares fit of the data, r^2^ values are displayed in the plots. For all plots, the blue square is the control group; the green diamond, single-agent carboplatin group; yellow triangle, single-agent paclitaxel group; and red circle, carboplatin/paclitaxel treated tumours. In panel (d) the single-agent carboplatin and single-agent paclitaxel treatment groups are overlapped.

The total tumour area was calculated from both the histological images and ADC maps (Table 3). Tumours from the control group exhibited the highest cross-sectional area and the largest sum of the tumour axes. The combination carboplatin/paclitaxel treatment group displayed the smallest tumour areas. There was a moderate correlation (r^2^ = 0.74–0.90) between the cross-sectional size of the tumours, measured from the histological images, and the mean and maximum ADC values (Fig. 4a,c).

### Mechanical Testing

The elastic moduli of the tumour samples from all treatment groups were measured by unconfined compression testing and averaged over the given group. The results are shown in Table 4. For all treatment groups, the elastic moduli were higher than the elastic modulus for the control group. The strongest increase was observed for the combination carboplatin/paclitaxel treatment group. A strong correlation (r^2^ = 0.85–0.96) between the *inverse* of the elastic modulus and the mean as well as the maximum ADC was observed across the treatment groups (Fig. 4b,d).

### Gene Expression

The changes in the gene expression of ECM constituents for each of the treatment groups relative to the control group were evaluated (see Table 5). As the results were obtained from one tumour sample per group, we regard these changes only as a qualitative indication of an increase or decrease in gene expression. The combination carboplatin/paclitaxel treatment group displayed a consistent increase in the expression of all the ECM genes investigated, with an especially substantial increase in the expression of fibronectin (*FN1*).

## Discussion

The use of clinical imaging to monitor tumour response to therapy continues to rely largely on changes in uni-dimensional measures of the tumour size, evaluated according to accepted standards, such as Response Evaluation Criteria In Solid Tumours (RECIST)^37^,^38^. However, measureable changes to tumour size occur late in therapy and cannot reflect treatment-induced physiological changes. As a result, there is a growing need for the development of imaging biomarkers that detect earlier treatment-induced changes at both macromolecular and microstructural levels. The ADC is one potential imaging biomarker that can be implemented in both clinical and research settings. Diffusion of water in biological tissues is affected by a range of intra- and extracellular characteristics, including cell density, morphology, membrane permeability and ECM composition. At high cell densities, these effects are amplified^7^,^39^, and the measured ADC is highly susceptible to changes in the tumour microenvironment. Thus, the ADC is well-suited for quantitative evaluation of tumour responses to therapy. The development of functional imaging biomarkers, such as the ADC, is particularly important for ovarian cancer, where the application of RECIST can be difficult^40^. Despite this, the literature on changes to ADC values and comparative analysis of the ADC distributions in ovarian lesions as a result of treatment are limited.

### ADC in Ovarian Cancer

The potential of the ADC as an imaging biomarker for the detection and staging of ovarian cancers has been studied more widely than the use of the ADC as a tool for monitoring tumour response to therapy^13^,^14^,^41^,^42^,^43^,^44^,^45^,^46^,^47^. However, there have been varying findings as to the usefulness of the ADC in regard to the former due to the broad overlap between the ADC distributions of benign and malignant lesions. This overlap is a result partly of the variation of the anatomy and the progression of the tumours, and partly a result of the existence of different tumour subtypes^13^,^43^,^47^. The OV-MZ-6 cells used in the present study to generate the tumour xenografts were established from a patient diagnosed with advanced serous cystadenocarcinoma (FIGO IV) of the ovary^48^. The reported mean ADC values measured in serous adenocarcinomas from clinical studies are displayed in Table 6. In the present study, the mean ADC was (0.53 ± 0.06) × 10^−3^ mm^2^ s^−1^ for the control group (no treatment); this is substantially less than the values reported previously in patient-derived tissues. This is not surprising, given the advanced stage (FIGO IV) of the tumour xenografts in the present study combined with research-imaging conditions. Lower mean ADC values have been linked to higher FIGO stages in serous adenocarcinomas^41^. The other factor responsible for the differences between the present and previously reported mean ADC values is that the diffusion measurements in the present study were performed at 25 °C, which corresponds to the bulk-water diffusion coefficient ~30% lower than at the standard physiological temperature (37 °C)^49^,^50^.

### Sample Size

The intraperitoneal ovarian cancer animal model used and the number of animals per group are consistent with the protocol established by our group previously^33^. The animal study was conducted using 5 NOD/SCID mice per group over 8 weeks, with treatment (control, carboplatin, paclitaxel, carboplatin/paclitaxel) starting in week 4 post implantation of spheroid-loaded hydrogels. A similar sample size has been demonstrated to provide statistically reliable results in our previous studies^33^.

The inter-group differences in the response to treatment explain the different numbers and sizes of the tumour samples available for the DW-MRI measurements and mechanical testing from each treatment group. From the carboplatin and the control groups, which were less responsive to treatment, three and five samples, respectively, were recovered for subsequent analysis; these samples exhibited a relatively large size (see Table 3). From the more responsive paclitaxel-treated groups, two and five samples, respectively, were available; the sizes of the samples were relatively small. Due to the surrounding murine tissue, only clearly identifiable tumour areas were included in the DW-MRI analysis, as indicated in the FLAIR images (see Fig. 2). Despite the differences in the number and the size of the samples obtained from different groups, the tumour samples in each group originated from the same number of animals. As a result, the MRI measurements essentially compared the combined tumour burden of the five animals present in each group, and therefore the intergroup comparison was not biased by the differences in tumour sample sizes.

### ADC Changes in Response to Treatment

The mean ADC values exhibited an increase in the treated tumours compared to control (no treatment) tumours. The combined mean ADC of the treated tumours was on average 7.5% larger than that of the control. Similar increases in the mean ADC have been observed in clinical studies of patients with ovarian cancer treated with different combination platinum/taxane regimens as displayed in Table 6 30,31,51. An increase in the ADC values in treated tumours is consistent with an increase in extracellular space following cell death through various mechanisms^52^. In ovarian cancer, carboplatin and paclitaxel are known to induce apoptosis^53^, which has been directly linked to increases in the ADC^23^,^52^,^54^. While the ADC values increased in all treatment groups compared to the control, this change was not equal across the distribution of the ADC. Kyriazi *et al*.^30^ have also demonstrated that changes to ADC distributions in ovarian cancers in response to carboplatin- or paclitaxel-based treatments are heterogeneous. The spatial heterogeneity of tumour responses to treatment is an ongoing problem in the development of the ADC as an imaging biomarker for tumour responses to therapy^55^,^56^,^57^. The variation in tumour response may be due to spatial heterogeneity in the pre-treatment tumour tissues. In addition, changes to the ADC in response to treatment are also heterogeneous. In the present study, the variation of tumour response to treatment was observed separately in all the treatment groups tested, and was dependent on the treatment regimen.

### Changes in Tumours Treated with Single-Agent Carboplatin or Paclitaxel

The results of the Mann-Whitney test demonstrate that the ADC distributions of the single-agent carboplatin and paclitaxel treatments group were significantly different (Table 2). The mean ADC in the tumours treated with single-agent carboplatin or paclitaxel was 6% and 9% larger, respectively, than in the control. The difference in the change in the minimum ADC from the control group was more pronounced for the two single-agent treatment groups. The minimum ADC value (Table 1; defined as the first percentile of the ADC distribution) of the single-agent carboplatin treated group was within 2.5% of the minimum ADC value in the control group. However, the minimum ADC value for the single-agent paclitaxel treatment regimen was ~20% larger than the control. This suggests that the single-agent paclitaxel was more effective than single-agent carboplatin in inducing cell death in regions of higher cell density in the tumour xenografts, and resulted in the lower number of proliferating cells. Conversely, the maximum ADC value (Table 1; defined as the 99^th^ percentile of the ADC distributions) was the same for both single-agent treatment regimens.

### Changes in Tumours Treated with Combination Carboplatin/Paclitaxel

The tumours treated with combination carboplatin/paclitaxel were smaller in size, revealed the lowest number of proliferating cells and exhibited substantial increases in ECM constituents compared to the single-agent treated tumours. These observations indicate that the combination regimen was the most successful of the chemotherapy regimens investigated. Changes in ECM constituents have been linked to chemo-resistance in ovarian tumours^58^, and increased fibronectin expression has been associated with cisplatin chemo-resistance in other cancer types^59^. Thus, increases in ECM constituents during treatment can be considered the tumour’s survival response to treatment and an indicator of success or failure of chemotherapy.

There was no statistically significant difference between the mean ADC of the tumours treated with single-agent paclitaxel and the combination carboplatin/paclitaxel. Additionally, the minimum ADC values of both the single-agent paclitaxel and combination carboplatin/paclitaxel treatment groups were the same. This suggests that the difference between the lower portions of the ADC distribution curves (which correspond to the regions of tumours with relatively high cellularity) between the control and the combination carboplatin/paclitaxel treatment groups can be attributed to paclitaxel.

In addition to changes in cell density, the ADC is sensitive to morphological and compositional changes in the ECM of tumour tissues. The increase in ECM constituents introduces additional obstructions and hydrogen-bonding sites to the tumour microenvironment, reducing the ADC. It is often observed, usually with reference to fibrosis, that later stages of chemotherapy may induce changes in the ECM of tumours that affect the ADC^3^,^18^,^57^. The sensitivity of the ADC to changes in the ECM suggests that interpretation of changes in the measured ADC in terms of treatment response at later time periods should include changes to ECM composition in addition to cell density.

It is not clear whether the magnitude of the increase in ECM proteins in the present study is sufficient to directly impact the measured ADC in the tumours treated with the combination carboplatin/paclitaxel. To investigate this further, we propose that imaging at multiple, earlier time points should be conducted in future studies. This would not only allow the changes to the ADC to be monitored throughout different stages of ECM production, but also provide insight into the time dependence of ECM production in the tumour xenografts as a survival or apoptosis response to chemotherapy. This would enable the relationship between the measured ADC distribution and the molecular and cellular response of the tumours to therapy to be understood in greater detail.

The minimum and the mean ADC values exhibited no significant differences between combination carboplatin/paclitaxel and single-agent paclitaxel treatment groups. Despite this, the maximum ADC for the combination carboplatin/paclitaxel treatment was ~8% larger than in the single-agent paclitaxel treatment group. Higher ADC values correspond to regions of lower cell and ECM density, making the ADC in these regions less susceptible to changes in ECM composition. Therefore, at later stages in chemotherapy, during ECM compositional or morphological changes, the maximum ADC value may provide a better indication of treatment efficacy in terms of cell death alone.

### Distribution of the ADC across the Tumour ROIs

Table 1 shows that, in any given treatment group, the ADC of the tumour tissue exhibits significant variations across the tumour region-of-interest. A useful indicator of the magnitude of these variations is the ratio of the 99^th^ percentile (“the maximum ADC”) to the 1^st^ percentile (“the minimum ADC”) of the ADC distribution; this ratio ranges between 1.54 for the paclitaxel-only group and 1.87 for the carboplatin-only group. The ADC distribution parameters shown in Table 1 also mean that the maximum ADC can be up to 32% larger than the corresponding mean ADC, while the minimum ADC can be as much as 33% lower than the mean ADC. ADC variations of this magnitude are not unusual: comparable or even larger ADC variations are routinely observed in other studies, including *in vivo*^30^. This can be attributed to at least two principal factors. First, the measurement of the ADC is subject to noise, which means that even in a hypothetical perfectly uniform tissue the distribution of the ADC values measured in different voxels would exhibit a non-zero variance. The second factor is the heterogeneity of the tumour tissue, i.e., the inevitable variations in the cell density, vascularity, the composition and the density of the ECM and other morphological parameters across the ROI. As discussed above, these morphological factors affect the value of ADC, and their variation across the tumour ROI leads to additional variance of the ADC distribution. Separation of the ADC variance attributable to noise and to tissue heterogeneity would require a careful and technically demanding experimental design: for example, an accurate measurement of the true instrumental noise would likely require the use of highly uniform, carefully designed phantoms in order to exclude the effects of tissue heterogeneity. We have not attempted such separation in the present study; nor are we aware of attempts in other studies to separate the different contributions to ADC variance in tumours. Nevertheless, a number of observations suggest that ADC variations due to tumour heterogeneity are an important indicator of the status of the tumour and its response to treatment. In the present study, we have observed a stronger correlation between the maximum ADC values and the stiffness of the tumour than between the mean ADC and tumour stiffness (see the discussion below). Other authors have observed treatment-induced changes in the shape of the ADC distribution that differ between responders and non-responders^30^. The same authors have found that, based on the area under receiver operating characteristic curve (AUC) test, the relative change of the 25^th^ ADC percentile was a better predictor of the response to treatment than either the median ADC or the shape-based characteristics of the ADC distribution such as skewness and kurtosis. Recent experimental studies of breast tumours *in vitro* combined with diffusion modelling^60^ have found that MRI diffusion measurements of tumours are significantly influenced by the effects of restricted diffusion and diffusion anisotropy. Both of these factors can result, independently of noise or tissue heterogeneity, in the presence of a distribution of ADCs as well as other diffusion-related characteristics; furthermore, the nature of the distribution can be strongly dependent on the nature of the model used to interpret the diffusion data. All these findings suggest that a hypothetical structureless, morphologically uniform tumour is not a good model to use in the interpretation of ADC measurements, and the entire ADC distribution (or, more generally, the distribution of restricted-diffusion parameters) should be used when evaluating the response of the tumour to drug treatment on the basis of diffusion measurements.

### ADC and Tumour Area

The idea that high-ADC regions are particularly indicative of treatment-induced cell death is reinforced by the correlation between the maximum ADC and tumour size. Of the different area measures reported in Table 3, the histological area, identified by NuMA-positive staining, is the most representative of the total tumour region. The maximum ADC showed a closer correlation with the cross-sectional area of the tumour samples, measured by histology, than the mean ADC values.

### ADC and Elastic Modulus

There was a strong negative correlation (r^2^ = 0.96; Fig. 4d) between the maximum ADC values and the *inverse* of the elastic modulus. This is an apparently counterintuitive behaviour, which suggests that the treatment-induced reduction in tumour tissue (increasing ADC) was accompanied by an increase in the mechanical stiffness (decreasing 1/Young’s modulus). Previous studies have reported a decrease in shear stiffness as a result of anti-cancer treatment^61^,^62^; and increased stiffness in tumour tissue compared to normal tissue has been seen in various tumour types^63^,^64^,^65^. Nevertheless, treatment-induced increase in the mechanical stiffness of the tumour tissue has been observed in past tumour xenograft studies^36^. The strong correlation between the maximum ADC values and the Young’s modulus of the xenografts (Fig. 4d) suggests that this increase has a biophysical basis in the tumour microenvironment and the response of this microenvironment to drug treatment. Investigation of changes in tumour stiffness in response to treatment is still a relatively new field of research, and a number of factors not yet understood could play a role. Stiffness in biological tissues is dependent on both morphological and compositional characteristics of the ECM, as well as cell stiffness. Increased stiffness in tumour tissue can be a result of an upregulation of the production of fibrous proteins, and the consequent formation of ECM structures that are larger, more rigid and more disordered than those in normal tissues^66^,^67^. The combination carboplatin/paclitaxel treatment group had the largest elastic modulus (~6 times greater than the control) and a substantial increase in ECM constituents, suggesting that it is the density of the ECM (as opposed to cellular density) that could have been responsible for the observed stiffness increase in the treated tumours. Further investigations of the quantitative relationship between these factors would be of interest to cancer research.

### Limitations

The evaluation of the response to treatment in the present study was based on a comparative statistical analysis of the ADC distributions obtained from different treatment groups. The key statistical tool used, Mann-Whitney test, enables the detection of subtle changes in the ADC distributions; however, this test relies on the number of imaging voxels in the tumour ROIs being sufficiently large. At the same time, decrease in tumour area and the number of the tumours (and the accompanying reduction of the tumour ROIs) are unavoidable consequences of a successful treatment. Therefore, samples in the control group, or in groups with less effective treatments, will tend to have a larger tumour ROI (and more tumour voxels available for statistical analysis) than samples in the more responsive treatment groups. This was very apparent in the combination carboplatin/paclitaxel treatment group: only two samples were available for imaging, and of these, only one contained a solid tumour region. Therefore, the accuracy of the foregoing Mann-Whitney statistical analysis is inherently negatively biased in the case of successful treatment groups compared to the control and less successful treatment groups, single-agent paclitaxel or carboplatin, for which three and five samples, respectively, were available for testing. In future studies, the number of animals per group should be at least doubled in order to allow for a sufficient number of tumours (*n* ~ 5) in the more successful treatment regimens for DW-MRI measurements and ADC analysis.

## Conclusions

We have demonstrated that the distribution of the ADC and the maximum ADC value are well-suited for evaluating the response to chemotherapy in ovarian tumour xenografts. Changes in the ADC distribution in response to therapy varied and were dependent on anti-cancer agent. Treatment containing paclitaxel as single-agent induced changes in the high-cellularity regions of the tumour xenografts. The tumour area and the ECM gene expression analyses indicated that the combination carboplatin/paclitaxel-based regimen was more effective than the single-agent treatments. However, there was no distinction between the mean ADC of these tumour xenografts and the single-agent paclitaxel, indicating that the response of the tumour xenografts was majorly attributed to paclitaxel. We hypothesise that the reduction in ADC in the combination treatment group is a result of increased biosynthesis of the ECM constituents and recommend imaging different time points during treatment for future investigations. Such a study would also provide insight into the time dependence of ECM production in the tumour xenografts, leading to a better understanding of the role of ECM synthesis as a survival or apoptosis mechanism induced in chemotherapy.

## Additional Information

**How to cite this article:** Tourell, M. C. *et al*. The distribution of the apparent diffusion coefficient as an indicator of the response to chemotherapeutics in ovarian tumour xenografts. *Sci. Rep.* **7**, 42905; doi: 10.1038/srep42905 (2017).

**Publisher's note:** Springer Nature remains neutral with regard to jurisdictional claims in published maps and institutional affiliations.

## Figures and Tables

**Table 1:** ADC distributions for the different treatment groups corresponding to the ROIs shown in Fig. 2.

Group	Mean ± std. dev. (×10^−3^ mm^2^ s^−1^)	Min. ADC (×10^−3^ mm^2^ s^−1^)	Max. ADC (×10^−3^ mm^2^ s^−1^)
Control	0.53 ± 0.06	0.39	0.67
Carboplatin	0.56 ± 0.07	0.38	0.71
Paclitaxel	0.58 ± 0.06	0.46	0.71
Carboplatin/Paclitaxel	0.58 ± 0.06	0.47	0.77

The mean ADC value was equal to the median ADC value in the ADC distributions of all groups. The minimum and maximum ADC values were calculated as the 1^st^ and 99^th^ percentiles, respectively.

**Table 2:** Pairwise Mann-Whitney testing of the different groups.

Group	Control	Carboplatin	Paclitaxel	Carboplatin/Paclitaxel
Control	—	3.4 × 10^−14^	5.5 × 10^−45^	3.5 × 10^−20^
Carboplatin	3.4 × 10^−14^	—	2.0 × 10^−11^	4.7 × 10^−5^
Paclitaxel	5.5 × 10^−45^	2.0 × 10^−11^	**—**	**0.3**
Carboplatin/Paclitaxel	3.5 × 10^−20^	4.7 × 10^−5^	**0.3**	—

Each treatment group was found to be significantly different from the control group (no treatment). Bolded values indicate no statistically significant difference between the two distributions at α = 10^−4^.

**Table 3:** Total tumour area calculated in both the histological and MRI analyses.

Group	Histological area (mm^2^)	MRI area (mm^2^)	Sum of the longest axes MRI (mm)
Control	63 ± 14	52 ± 7	19.9 ± 0.8
Carboplatin	21 ± 9	29 ± 6	15.2 ± 0.8
Paclitaxel	32 ± 11	18 ± 3	10.2 ± 0.6
Carboplatin/Paclitaxel	11 ± 6	6 ± 1	3.7 ± 0.3

**Table 4:** The Young’s modulus of the different treatment groups averaged over the number of tumour tissue samples tested.

Group	Number of samples	Mean ± std. dev. (kPa)
Control	5	0.9 ± 0.4
Carboplatin	3	2.3 ± 1.4
Paclitaxel	5	2.4 ± 1.0
Carboplatin/Paclitaxel	2	6.1 ± 0.7

**Table 5:** Relative gene expression levels of different ECM proteins normalised to the control (no treatment) group (mean ± standard deviation).

Group	*FN1*	*VTN*	*COL1A1*	*HIF1A*
Control	1.00 ± 0.06	1.00 ± 0.11	1.00 ± 0.02	1.00 ± 0.04
Carboplatin	2.68 ± 0.29	2.18 ± 0.06	0.89 ± 0.04	1.87 ± 0.07
Paclitaxel	1.35 ± 0.03	0.72 ± 0.17	1.02 ± 0.02	0.72 ± 0.02
Carboplatin/Paclitaxel	20.04 ± 0.38	2.49 ± 0.28	3.01 ± 0.05	3.47 ± 0.06

**Table 6:** Mean ADC values in serous ovarian carcinoma determined in patient-derived tissues and in the present study.

Study	Ovarian tumour subtype (FIGO or number of patients or lesions)	Mean ± std. dev. ( × 10^−3^ mm^2^ s^−1^)
Fujii *et al*.^13^	serous adenocarcinoma (n = 19)	1.32 ± 0.31
Zhang P *et al*.^43^	serous adenocarcinoma (n = 35)	0.97 ± 0.20
Zhang H *et al*.^14^	serous adenocarcinoma (n = 12)	1.12 ± 0.42
Oh *et al*.^41^	serous adenocarcinoma (FIGO I)	1.17 ± 0.36
	serous adenocarcinoma (FIGO II-IV)	0.86 ± 0.15
Kyriazi *et al*.^51^	serous adenocarcinoma (FIGO III/IV), pre-treatment	0.90 ± 0.12
	serous adenocarcinoma (FIGO III/IV), post-treatment (first cycle)	0.99 ± 0.13
	serous adenocarcinoma (FIGO III/IV), post-treatment (second cycle)	1.05 ± 0.14
Kyriazi *et al*.^30^	serous papillary adenocarcinoma (n = 32), pre-treatment	1.08 ± 0.18
	serous papillary adenocarcinoma (n = 32), post-treatment (first cycle)	1.22 ± 0.22
	serous papillary adenocarcinoma (n = 26), post-treatment (third cycle)	1.30 ± 0.24
Sala *et al*.^31^	serous adenocarcinoma (n = 9), pre-treatment	1.02 ± 0.19
	serous adenocarcinoma (n = 9), post-treatment (third cycle)	1.30 ± 0.29
The present study (measured *in vitro* at 25 ^o^C)	serous adenocarcinoma xenografts (FIGO IV), no treatment	0.53 ± 0.06
	serous adenocarcinoma xenografts (FIGO IV), post-treatment	0.58 ± 0.06

If available, mean ADC values pre- and post-treatment are listed from patients responding to chemotherapy. All the measurements from the literature were made at the physiological temperature *in vivo* (~37 ^o^C); the measurements in the present study were made *in vitro* at 25 ^o^C.
